# IUGR Management: New Perspectives

**DOI:** 10.1155/2014/620976

**Published:** 2014-12-09

**Authors:** N. Giuliano, M. L. Annunziata, S. Tagliaferri, F. G. Esposito, O. C. M. Imperato, M. Campanile, M. G. Signorini, A. Di Lieto

**Affiliations:** ^1^Department of Public Health, University of Naples Federico II, Via Pansini 5, 80131 Naples, Italy; ^2^Politecnico di Milano, Piazza Leonardo da Vinci 32, 20133 Milan, Italy

## Abstract

*Aim of the Study*. Analyzing velocimetric (umbilical artery, UA; ductus venosus, DV; middle cerebral artery, MCA) and computerized cardiotocographic (cCTG) (fetal heart rate, FHR; short term variability, STV; approximate entropy, ApEn) parameters in intrauterine growth restriction, IUGR, in order to detect early signs of fetal compromise. *Population Study*. 375 pregnant women assisted from the 28th week of amenorrhea to delivery and monitored through cCTG and Doppler ultrasound investigation. The patients were divided into three groups according to the age of gestation at the time of delivery, before the 34th week, from 34th to 37th week, and after the 37th week. Data were analyzed in relation to the days before delivery and according to the physiology or pathology of velocimetry. Statistical analysis was performed through the *t*-test, chi-square test, and Pearson correlation test (*P* < 0.05). Our results evidenced an earlier alteration of UA, DV, and MCA. The analysis between cCTG and velocimetric parameters (the last distinguished into physiological and pathological values) suggests a possible relation between cCTG alterations and Doppler ones. The present study emphasizes the need for an antenatal testing in IUGR fetuses using multiple surveillance modalities to enhance prediction of neonatal outcome.

## 1. Introduction

According to ACOG guidelines, a fetus with intrauterine growth restriction (IUGR) is a fetus with an estimated weight less than the 10th percentile for gestational age [1]. With a prevalence of the 5–8% in the general population, IUGR can complicate 10% to 15% of all pregnancies [2].

Frequently the etiology of IUGR is unknown; however in several cases it is possible to identify fetal (infection, malformation, and chromosomal aberration [3]), placental [4] (chorioangioma, infarction, circumvallated placenta, confined placental mosaicism, obliterative vasculopathy of the placental bed, etc.), maternal (chronic hypertension [5], pregestational diabetes, cardiovascular disease [6], substance abuse, autoimmune conditions, etc.), and external factors that modulate the normal fetal growth, by acting on a genetically predetermined potential growth [7].

IUGR represents the second cause of perinatal mortality, after prematurity, and it is related to an increased risk of perinatal complication as hypoxemia, low Apgar scores, and cord blood acidemia, with possible negative effects for neonatal outcome [8, 9].

Liver perfusion is reduced to 30% [10, 11] so that the low fetal body weight can be partially caused by impairment of liver protein biosynthesis [12]. This diversion of oxygenated blood to preferential perfusion of vital organs such as the brain, heart, adrenal glands, and spleen [13–16] and reduced flow to less important organs such as muscles, bowel, and kidneys enables the fetus to survive for a considerable period. If the oxygen supply to the myocardium reaches its limit, the myocardium stiffens, and the central venous pressure increases [17].

Hemodynamic changes involve maternal uterine, fetal umbilical (UA), and middle cerebral (MCA) arteries and precordial veins for cardiac effects of placental dysfunction [18, 19]. The circulatory adaptation consists in an increased UA and decreased MCA blood-flow resistance [20].

Doppler investigation is an efficient method of surveillance in IUGR monitoring [21]. The relationship between UA and neonatal outcome is controversial [22, 23].

MCA was found to be a better predictor for fetal outcome in IUGR when compared with umbilical artery in terms of sensitivity and predictive value [24]. Instead, ductus venosus was considered as the strongest Doppler predictor of perinatal mortality in preterm IUGR fetuses [25–27].

Nevertheless, the use of Doppler velocimetry in cases of IUGR, although well studied, is still controversial and standardized guidelines are lacking. Therefore, Doppler ultrasound has to be integrated with several techniques of screening for a complete clinical evaluation of IUGR. Some authors [28] found out that intrapartum fetal Doppler velocimetry, when combined with cardiotocography (CTG), increases the clinicians' ability to accurately identify fetal hypoxia. In the last years, computerized cardiotocography (cCTG) has conquered an important role in medical management of pregnancy, especially in high risk patients. cCTG monitoring consists in the electric recording of fetal heart rate (FHR) and can be considered the most widespread noninvasive method to evaluate fetal well-being during prenatal and intrapartum process. cCTG offered a standardized method to evaluate conventional CTG parameters and introduced quantitative measures of linear and nonlinear indices related to FHR generation as a multiparametric analysis of fetal cardiovascular and nervous activity [29]. The presence of significant beat-to-beat variation suggests intact sympathetic/parasympathetic tone and central control indicating normal central nervous system (CNS) responsiveness and normal local CNS metabolic environment reflecting fetal health [30, 31].

Despite the fact that cCTG is widespread, its use is still thwarted because computer programs are considered inevitably based on the current, limited knowledge of fetal heart rate patterns, in relationship to neonatal long-term outcome [32]. For Baschat et al. [33], Doppler indices have a more important and statistically significant relationship with perinatal outcome [32, 33].

Since many authors [34, 35] have showed that Doppler velocimetry cannot be able, alone, to manage IUGR fetuses, we performed this retrospective longitudinal study based on a multiparametric analysis. Our aim was to evaluate the modifications of velocimetric (UA, DV, and MCA) and computerized cardiotocographic (FHR, STV, and ApEn) parameters in relationship to “days before delivery,” in order to find out those associated with earlier fetal compromise in fetal growth restriction.

## 2. Materials and Methods

This retrospective longitudinal study was carried out at the Public Health of Federico II University of Naples (Italy) in a period of five years (2008–2013).

The study was conducted on a sample of 375 pregnant women assisted from the 28th week of amenorrhea to delivery. Gestational age was accurately established or confirmed from ultrasound measurement of the embryo or fetus in the first trimester [36]. The diagnosis of IUGR was based on the evaluation of an abdominal circumference below the 10th percentile.

Inclusion criteria were Caucasian ethnic, singleton pregnancies, absence of preexisting maternal disease, and neonatal weight below the 10th percentile for the gestational age (weight evaluated according to nomograms by WHO, November 1, 2009). Antenatal examinations included ultrasound biometry, Doppler velocimetry on UA, MCA, DV, and antenatal cCTG monitoring.

Newborn baby data (sex, weight, Apgar score, malformation at birth, access to neonatal intensive care, and umbilical artery pH) were collected.

cCTG records were obtained using Corometrics 170, General Electrics. The cardiotocograph is equipped with two transducers: the first one is an ultrasound transducer to detect the fetal heart rate (FHR), posted next to the focus of maximum auscultation of fetal heart; the second one is a pressure transducer for uterine contractile activity located next to the uterine fundus.

The cardiotocograph is connected to a smartphone that, via general packet radio service (GPRS), sends traces to the operation center, interfaced to 2CTG2 system (SEA, Italy) for computerized analysis [37]. The following cCTG parameters, fetal heart rate (FHR), short term variability (STV), and approximate entropy (ApEn) were examined [38]. We considered a fetal heart rate <110 or >160 bpm [39], a short-term variability <5th percentile for gestational age [40], and ApEn <5th percentile [41, 42] abnormal.

Doppler evaluation was performed using a Toshiba Ultrasound Nemio XG with a 3.5–5 MHz curvilinear transducer for transabdominal examination and a 3.75–3.8 MHz transducer for transvaginal evaluation. Ultrasonography was performed in each pregnant woman and the insonation by the pulsed Doppler examination was improved with colour Doppler images to obtain velocity waveform for UA, MCA, and DV. Pulsatility index (PI) for each vessel was obtained and evaluated. PI of UA was considered abnormal when it was >97.5th percentile for gestational age [43], as well as when diastole was absent or reversed. Absent/reverse A-wave in DV [20, 34] and brain sparing in MCA were also detected [24, 44].

To discriminate between early and late fetal compromise, the study population was divided into three groups according to the gestational age of delivery (<34th; from 34th to 37th gestational week; >37th gestational week at time of delivery) and data were analyzed as a function of days before delivery. 24 hours was the time interval between Doppler alterations (ductus venous waveform or umbilical artery PI >95th centile; absent or reverse A-wave or end-diastolic flow in DV and in UA, resp., MCA PI less than the 5th centile) and CTG abnormalities (see criteria ACOG classification 2009 [45]).

Data statistical analysis was performed using version 18.0 SPSS for windows statistical package.

*t*-test with the Bonferroni adjustment was applied for continuous variables while chi-square test with the Bonferroni adjustment was used for categorical variables.

*t*-test investigated the existence of a statistical significant difference between the three groups for cCTG (FHR, STV, and ApEn) and Doppler velocimetric (UA, MCA, and DV) parameters. Moreover, among patients of each group, each parameter was related to the gestational age using the Pearson correlation test. To complete our analysis, patients were also divided according to the physiological or pathological Doppler indices and, also in these groups, cCTG parameters were analyzed through the *t*-test also in these groups.

Statistical significance with Bonferroni's correction was *P* value < 0.016.

## 3. Results

In our study, 98% of women who delivered before the 34th week of gestation had a cesarean section. This value was similar to the percentile reported in the TRUFFLE study [46]. Fetal pH at birth and the Apgar score were both in the range of normality (Table 1).

*t*-test with Bonferroni correction revealed a significant difference for* maternal age* between “<34th week” and “from 34th to 37th week” groups and between “<34th week” and “from 37th week” groups for* maternal age* and between each group of study compared to each one of the other two groups for* fetal pH*  (*P* < 0.016). Chi-square test with Bonferroni correction showed a significant difference for the* way of delivery*,* for Apgar value at 3 minutes,* and for the* gender* of newborns for each group compared to the other ones. For* Apgar <7 at 5 minutes* only between “<34th week” and “from 34th to 37th week” and between “from 34th to 37th week” and “>37th week” groups a difference was found (*P* < 0.016).

**Figure 1:**
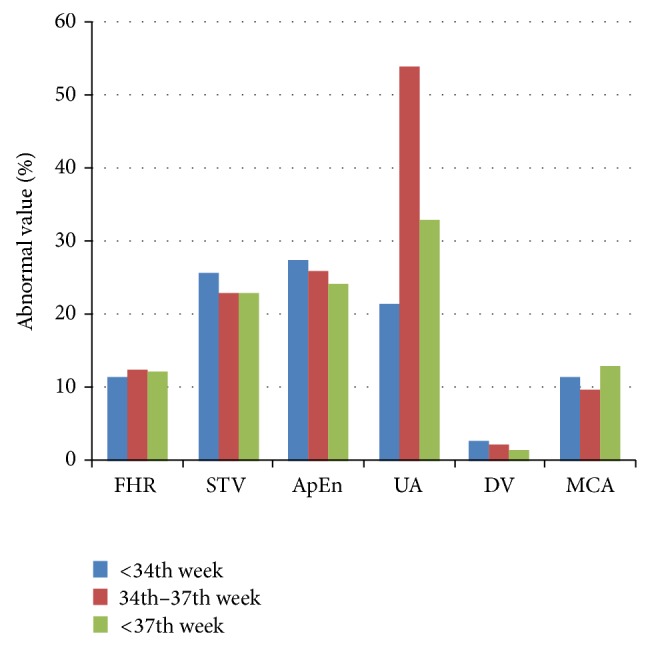
percentile of abnormal values in patients. UA, DV, and MCA are expressed as PI, pulsatility index.

Figure 1 shows the percentile of abnormal values for cCTG parameters and for Doppler indices. Chi-square test with Bonferroni correction evidenced a statistical significant difference between each group of study compared to each one of the other two (“before the 34th week” versus “from 34th to 37th week”; “before the 34th week” versus “after the 37th week”; “from 34th to 37th week” versus “after the 37th week” groups) for FHR, MCA, UA, and DV (*P* < 0.016). The only exceptions were found for STV and ApEn. In particular, STV was found different only between “<34th week” and the other two groups, while no difference was found between the two groups of study >34th week. Instead, considering the physiology or pathology of velocimetry, a statistical significant difference for each of cCTG indices (STV *P* = 0.002; ApEn *P* = 0.002) except for FHR (*P* = 0.03) was found.

**Figure 2:**
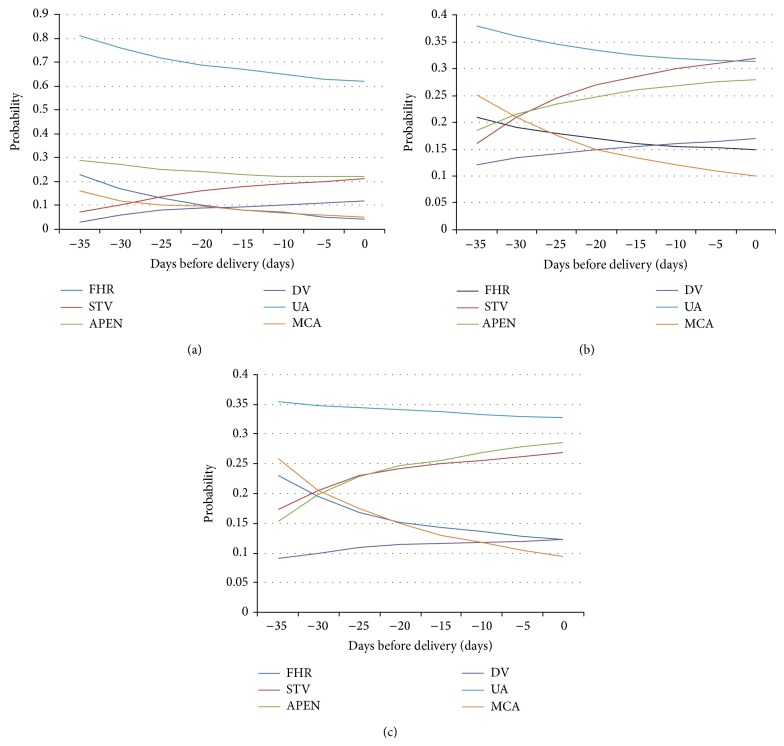
Probabilities of abnormal findings of variables in relation to time before delivery for (a) fetuses delivered before the 34th week of gestation. (b) Fetuses delivered between 34th and 37th week of gestation. (c) Fetuses delivered after the 37th week of gestation.

Figure 2 represents the trend of parameters in the three groups of study during pregnancy until the time of delivery expressed as the probability of finding a pathological value for each gestational age. STV and DV showed the earliest and most important modifications, while UA alterations were more marked only in the “<34th week” group.

In particular, among patients who delivered before the 34th week, Pearson correlation reported a decrease of each parameter except for STV and for DV. The correlation was statistically significant for FHR (*r* = −0.47; *P* = 0.021), MCA (*r* = −0.521; *P* = 0.002), DV (*r* = −0.721; *P* < 0.002), STV (*r* = 0.51; *P* = 0.0001), and ApEn (*r* = −0.41; *P* = 0.035). The only exception was found for UA (*r* = 0.073; *P* = 0.84).

For patients who delivered from the 34th to the 37th gestational age, the Pearson test showed significant correlations for FHR (*r* = −0.53; *P* = 0.015), MCA (*r* = −0.47; *P* = 0.01), DV (*r* = −0.49; *P* = 0.002), STV (*r* = 0.63; *P* = 0.002), and ApEn (*r* = −0.53; *P* = 0.024). UA is an exception (*r* = −0.2; *P* = 0.76).

For patients who delivered after the 37th week, the Pearson correlation showed a decrease for each parameter, except for STV. However, only for FHR (*r* = −0.51; *P* = 0.002), MCA (*r* = −0.436; *P* = 0.01), and DV (*r* = −0.52; *P* = 0.002) the correlation was statistically significant. For STV (*r* = 0.073; *P* = 0.67), ApEn (*r* = −0.01; *P* = 0.96), and UA (*r* = −0.18; *P* = 0.331) the modifications were not significant.

## 4. Discussion

This study was performed to improve the management of IUGR fetuses by integrating Doppler ultrasound evaluation with antepartum computerized cardiotocographic monitoring.

In particular, we evaluated the modifications occurring in hemodynamic and computerized cardiotocographic parameters as indicators of a progressive adaptation in a IUGR population. Our choice is based on the evidence that the cCTG is actually considered the most widespread noninvasive method of fetal well-being surveillance. On the other hand, Doppler ultrasound is a fundamental tool to evaluate IUGR fetus in relationship with fetal vascular abnormalities.

Decreased middle cerebral artery impedance and increased brain venous blood flow velocities characterize the brain sparing effect. These “early responses” are physiologically followed by late-onset Doppler abnormalities such as absent/reversed umbilical artery end-diastolic velocity, absent/reversed inferior vena cava and ductus venosus waves, and umbilical vein pulsation [16, 27, 33, 47–49]. In particular, the longitudinal progression of abnormal Doppler waveforms in the IUGR deterioration of uteroplacental function is the following: elevated umbilical artery blood flow resistance and reduced umbilical vein flow volume per kilogram of fetal body weight, both of which precede the onset of a growth delay [27, 50]. However, recently, Kessous et al. [34] have showed that UA and MCA measurements have a weak correlation with perinatal outcome, that means that a physician's decision regarding the management of a patient with suspected IUGR is complicated and influenced by several variables. To date, the relationship between Doppler and CTG monitoring parameters is still controversial. Kaponis et al. reported that alterations of venous flow volume waveforms precede fetal heart rate decelerations and therefore offer warning signs to act before a fetal life-threatening situation occurs [51]. For Baschat, instead, placental Doppler is the most powerful predictor of the clinical deterioration of IUGR fetus while biophysical abnormalities may not extend beyond loss of heart rate reactivity or the decrease in the amniotic fluid index [52, 53]. As for the time of delivery of IUGR at term, a previous observational study suggests that induction of labor is associated with an increased incidence of obstetric interventions, without any neonatal benefit. Instead, later randomized trials like DIGITAT show no effect of induction on adverse neonatal outcomes [54].

### 4.1. Doppler Velocimetry and cCTG Parameters: Our Results

Integrating Doppler velocimetry with the antepartum cCTG monitoring may be useful to manage pregnancies complicated by IUGR and especially could help the clinician's decision about the time of delivery. Our assumptions are based on the fact that both cCTG and Doppler parameters were found statistically different in the three groups of study divided according to the age of gestation at the time of delivery. Interestingly, we found that the three groups differ from each other also in the way of delivery, fetal pH at birth, and the Apgar values at 3 minutes. Finally, more important is that all the cCTG and Doppler parameters of the study have a significant correlation with the age of gestation, except for UA, before the 37th week (<34th week and from 34th to 37th week), and also for ApEn after the 37th week.

Approximate entropy, a mathematical approach to quantify the complexity of a system, consists in the clinical application of chaos theory. Previous studies [55, 56] had analyzed the relationship of ApEn with maturity of autonomous nervous system (ANS), thus emphasizing the relationship of a low value of ApEn with a lower Apgar score and metabolic acidosis. In our study, we found that ApEn progressively and significantly decreases in the <34th week group. Since these patients delivered more frequently through an urgent caesarean section, we hypothesize that they have a greater primitive fetal compromise or the fetal compromise could be a consequence of the deterioration of maternal conditions, and this compromise is evidenced by ApEn. With the progress of pregnancy, ApEn values increase, but with an even lower significance.

Probably, for a better evaluation of the differences in ApEn in the three groups, a further investigation on other complexity indices (sample entropy, multiscale entropy, the Lempel Ziv complexity, and detrended fluctuation analysis) would be needed. These parameters previously analyzed have not been introduced yet in the clinical routine management [57].

When comparing the cCTG parameters with flowmetric indices (distinguished into physiological and pathological ones), a significant difference was found. Unlike Ferrazzi et al., who had observed that over 50% of fetuses delivered for abnormal fetal heart rate patterns did not have Doppler abnormalities [58], we found, instead, that the abnormalities of cCTG parameters can be correlated with Doppler ones in growth restricted fetuses.

### 4.2. Temporal Trend of Modifications

The temporal trend of cCTG and Doppler parameters in relation to “days before delivery” was similar in the three groups of study, with an earlier alteration of UA, MCA, and DV, in comparison with the cCTG parameters, as reported by Baschat and Cosmi [53, 59]. These results were in contrast with those of PORTO study in which a predictable progressive sequence of Doppler deterioration was not found [60]. Probably, the disparity of results depends on the absence of stratification of population object of Porto study.

In our study, the PI of UA progressively decreased and it decreased more acutely among the two groups of patients who delivered before the 37th week. The more rapid decrease of UA-PI is consistent with a worse condition of these fetuses compared to those born after the 37th week. In fact, the UA waveform reflects placental alterations as the dimensions of the villous vascular tree, the blood flow resistance in the fetal compartment, and the relative risk for nutritional and metabolic deficiency [18, 53]. However, this evidence did not achieve the statistic relevance in all the three groups. Also MCA-PI progressively reduced in the whole population, showing that the brain sparing effect was not frequently present. Instead, this trend is consistent with a physiological decrease of the vascular resistance in the brain with advancing gestational age [61].

In growth restricted fetuses, abnormal ductus venosus is considered an excellent predictor of adverse perinatal outcome [58, 59] and a fundamental tool for choosing the optimum timing of delivery [62], as its abnormalities are typically associated with an increased risk for metabolic derangement or stillbirth [53, 63]. In our study DV-PI exhibited a trend towards a decrease, which was also statistically significant compared to the progression of pregnancy.

As regards the cCTG parameters we found fetal heart rate significantly reducing in the three groups of study [64]; physiological basis is a lower maturity of parasympathetic nervous system, in comparison with a normal FHR seen in normal growth fetuses [65]. A more marked reduction of FHR was observed in fetuses born after 37th week, maybe because of the increasing modulation by the cardiovascular function over the parasympathetic nervous system.

The probability of alteration of STV increased, especially few days before delivery, suggesting that STV reflects the more acute changes in fetal condition [63]. However, the relationship between this trend and the gestational age was statistically significant only in the two groups of patients who delivered before the 37th week. In fact, STV is considered as the best cCTG indicator of fetal ANS maturity, influencing not only the heart rate but also the vascular tone and the resistance cord [30].

### 4.3. Look at the Future

In our opinion, although we found important relationships between cCTG parameters and Doppler indices and between them and the gestational age at time of delivery in growth restricted fetuses, we still have concerns about how and when to intervene.

Thus, we think it is essential to detect new parameters to improve the IUGR management. An effort has been performed through the development of a new method for cardiotocographic signal analysis: the “phase-rectified signal averaging” (PRSA). It is based on the synchronization of the phase of all periodic components of the noisy, nonstationary signal [66]. PRSA analyzes all periodic components of the signal, irrespective of their frequencies or characteristic time scales, and it gives an approximate distinction of the separate effects of the vagal and sympathetic nervous system [67, 68]. To date, even if it characterizes rhythm modulations based on sympathetic activity, there is still poor evidence about the diagnostic power as a fetal surveillance method.

## 5. Conclusions

Studies evaluating the monitoring of pregnancies complicated by IUGR are greatly heterogeneous, partly because our understanding of its pathophysiology is actively evolving. As a consequence, worldwide accepted guidelines about fetal growth restriction monitoring are not available and the decision to deliver a preterm IUGR fetus still remains one of the great challenges in obstetrics. It is evident that IUGR fetuses with placental insufficiency require antenatal testing using multiple surveillance modalities to enhance prediction of neonatal outcome and birth pH [69].

Our study, driven by the necessity of a clear combined clinical evaluation, provides a first step to a serious consideration of cCTG monitoring and Doppler velocimetry together as tools to detect the time of delivery in IUGR fetuses before a life-threatening event can occur, achieving, at the same time, all the time possible to limit complications related to premature birth.

Certainly, there are questions still unanswered; for example, how could the indices of complexity be used in the clinical routine? What is the clinical role of less common cCTG parameters, such as the spectral analysis? And could the PRSA be a method of reconciliation between Doppler and cCTG supporters?

## Figures and Tables

**Table 1:** Maternal and perinatal characteristics.

Characteristics	<34th week	34th–37th week	>37th week
Basic demographic data			
Patients (%)	20.6	29.3	50.1
Maternal age (year)^1^	28 ± 4	28 ± 3	27 ± 2
Week of delivery (week)^1^	32.7 ± 1.85	36.43 ± 1.94	39.92 ± 1.97
Vaginal delivery (%)	2.1	6.2	4.1
Caesarean section (%)	97.9	93.8	95.9
Neonatal data			
Fetal pH at birth^1^	7.321 ± 0.061	7.322 ± 0.065	7.321 ± 0.075
Apgar <7 at 3 min (%)	19.51	13.35	12.24
Apgar <7 at 5 min (%)	7.22	6.37	0
Female (%)	41.63	38.42	47.96
Birth weight (g)^1^	1150.1 ± 245.63	1570.35 ± 265.31	1956 ± 330.46

^1^Values above are expressed as mean value ± standard deviation.
